# LINC01094/SPI1/CCL7 Axis Promotes Macrophage Accumulation in Lung Adenocarcinoma and Tumor Cell Dissemination

**DOI:** 10.1155/2022/6450721

**Published:** 2022-09-09

**Authors:** Zhuo Wu, Xue Bai, Zhengbo Lu, Shijun Liu, Hongfang Jiang

**Affiliations:** ^1^Department of Thoracic Surgery, Fourth Affiliated Hospital of China Medical University, Shenyang, Liaoning 110032, China; ^2^Department of Geriatrics, Shengjing Hospital of China Medical University, Shenyang, Liaoning 110004, China

## Abstract

**Objective:**

Infiltration of tumor-associated macrophages is closely linked to the malignant development of human cancers. This research studies the function of C-C motif chemokine ligand 7 (CCL7) in the macrophage accumulation in lung adenocarcinoma (LUAD) and the underpinning mechanism.

**Methods:**

The expression profile of CCL7 in LUAD and its correlations with patient's prognosis and macrophage infiltration were predicted via bioinformatics systems. Artificial up- or downregulation of CCL7 was induced in LUAD cells to explore its function in the mobility, EMT of cancer cells, and migration of M2 macrophages. Cancer cells were implanted in NOD/SCID mice to induce xenograft tumors. The CCL7-related transcription factors or factors were predicted by bioinformatic tools, and the molecular interactions were confirmed by immunoprecipitation or luciferase assays.

**Results:**

CCL7 was highly expressed in LUAD and linked to increased TAM infiltration. Knockdown of CCL7 suppressed the chemotaxis and M2 skewing of macrophages, and it blocked the EMT and mobility of LUAD cells. CCL7 downregulation also suppressed macrophage infiltration in xenograft tumors in mice. Spi-1 proto-oncogene (SPI1) was confirmed as an upstream factor activating CCL7 transcription, and LINC01094 was found to bind to SPI1 to promote its nuclear translocation. Upregulation of SPI1 restored the chemotactic migration and M2 polarization of macrophages in LUAD cells.

**Conclusion:**

This paper reveals that LINC01094 binds to SPI1 to promote its nuclear translocation, which further activates CCL7 transcription by binding to its promoter, leading to M2 macrophage accumulation and dissemination of tumor cells.

## 1. Introduction

Lung cancer takes a place of 11.4% among all cancer cases worldwide while accounting for 18% of all death cases, leaving it the leading contributor to cancer-related death [1]. Non-small-cell lung cancer (NSCLC) is the major subclass of lung cancer that makes up for ~85% of all cancer cases, and lung adenocarcinoma (LUAD) represents the major subtype of NSCLC [2, 3]. Despite progress in the diagnosis and therapy for LUAD, such as surgery, chemotherapy, radiotherapy, and immunotherapy, the treating outcome remains frustrating with the 5-year survival less than 20% [4]. In particular, owing to the lack of early symptoms, most patients are diagnosed at advanced stages with local of distant metastasis, whose survival rate is extremely low [5]. Developing more effect therapeutic options for lung cancer treatment remains a major task for researchers in this field.

The tumor microenvironment (TME), featured with the shortage of nutrients, hypoxic, and acidic environment, enriches with cancerous and noncancerous cells triggering tumor development, invasion, and dissemination [6]. Tumor-associated macrophages (TAMs) are highly abundant leukocytes in lung cancer whose mutual conversion is determined by the TME [7]. Upon tumor stimuli, TAMs shift toward to antitumor (M1) or a protumor (M2) phenotypes [8]. The M2-skewed macrophages represent the major infiltrating immune cells leading to tumor growth, epithelial-mesenchymal transition (EMT), metastasis, angiogenesis, and immunosuppression [7, 9], making them promising targets for antitumor treatment.

C-C motif chemokine ligand 7 (CCL7) is a chemotactic factor of the CC subfamily and an attractant of monocytes initially found in the culture supernatant of osteosarcoma cells [10]. CCL7 is lowly expressed in endothelial cells, fibroblasts, and monocytes and elevated upon a variety of stimuli such as interferons and viruses [11]. CCL7 deficiency in mice led to failed pathogen elimination, and the mice showed impaired neutrophil and monocyte infiltration in infected tissues or organs [12], suggesting its essential roles in recruiting immune cells to the infected microenvironment. Of note, CCL7 has been summarized to play critical prooncogenic role in cancers via a multitude of mechanisms, including the recruitment of immune cells to the tumor sites [13]. High CCL7 expression has been reported in NSCLC and linked to malignant phenotypes including the mobility and metastasis of cancer cells [14]. However, the functions of CCL7 in TAM infiltration in lung cancer and the underpinning mechanisms remain unclear.

In this work, the authors aimed to investigate the roles of CCL7 in the migration and polarization of macrophages and the malignant phenotypes of LUAD cells by performing gain- and loss-of-function assays. Moreover, we predicted the upstream regulators of CCL7 by using integrated bioinformatic analyzing tools and systems, and two candidate upstream regulators including LINC01094 and Spi-1 proto-oncogene (SPI1) were obtained. Collectively, a LINC01094/SPI1/CCL7 axis was defined in LUAD, which is potentially linking to macrophage infiltration and tumor development.

## 2. Materials and Methods

### 2.1. Reagents, Primers, and Antibodies

Enzyme-linked immunosorbent assay (ELISA) kits were procured from R&D Systems Inc. (Minneapolis, MN, USA), and the detailed information is presented in Table 1. The primers were procured from Sangon Biotech Co., Ltd. (Shanghai, China) with the sequence information given in Table 2. The antibodies used are listed in Table 3.

### 2.2. Bioinformatics Analysis

First, the expression of CCL7 in several LUAD datasets was retrieved in the Oncomine database (http://www.oncomine.org/) as well as in TCGA-LUAD (https://portal.gdc.cancer.gov/). Transcriptional factors in TCGA-LUAD that can bind with CCL7 promoter with an over 0.5 correlation coefficient were analyzed using the R TFBS package (http://bioconductor.riken.jp/packages/3.2/bioc/html/TFBSTools.html). We obtained 510 tumor samples from TCGA-LUAD and discarded over 80% nonexpressed genes with Log2 (TPM+1) as the thresholds. Putative binding between LINC01094 and SPI was predicted via the RPIseq system (http://pridb.gdcb.iastate.edu/RPISeq/).

### 2.3. Cell Treatment

The H1299, A549, and H358 cells (low-invasive) and H125 cells (invasive) were procured from ATCC (Manassas, VA, USA). All cells were cultured in RPMI-1640 (Thermo Fisher Scientific, Rockford, IL, USA) with 10% FBS. HEK-293T cells were procured from ATCC as well and maintained in 10% FBS-contained DMEM (Thermo Fisher Scientific). The condition for cell incubation was maintained at 37°C with 5% CO_2_. All cell lines used were free of mycoplasma infection.

Thereafter, 15-*μ*g short hairpin (sh) RNA of CCL7, the scramble plasmid, or overexpression plasmid of SPI1 or CCL7, 9 *μ*g psPAX2, and 6 *μ*g pMD2G package plasmid were mixed with 45 *μ*L Lipofectamine 2000 (Thermo Fisher Scientific) and transfected into HEK-293T cells. After 48 h, the lentiviral vectors were used to infect the H125 or A549 cells. After centrifugation using 8 *μ*g/mL (Sigma-Aldrich, Merck KGaA, Darmstadt, Germany) at 1,200× *g* at 32°C for 90 min, cells infected with lentivirus were harvested for functional analyses.

### 2.4. Quantitative PCR (qPCR) Analysis

DNA was amplified by DNA polymerase (Vazyme Biotech Co., Ltd, Nanjing, Jiangsu, China). The qPCR analysis was then performed using the TB Green® Premix Ex Taq™ (Takara Holdings Inc., Kyoto, Japan) on the Real-Time PCR system (Applied Biosystems, Foster City, CA, USA). Relative expression values of gene were calculated by the 2^-△△Ct^ method. GAPDH was used as the endogenous loading.

### 2.5. Immunoblot Analysis

Cells were lysed in RIPA buffer (Beyotime Biotechnology Co., Ltd, Shanghai, China). Total protein was collected and separated by 10% SDS-PAGE (EpiZyme, Shanghai, China) and loaded onto PVDF membranes (Millipore, Billerica, MA, USA). After incubation with the primary antibodies at 4°C for 16 h, the membranes were further incubated with horseradish peroxidase-labeled secondary antibody at 37°C for 1 h. The images were developed using the ECL HRP substrate (Millipore) and analyzed by ImageJ. Full scans of original gels were provided in Supplementary file 1.

### 2.6. Induction of M0 Macrophages Using THP-1 Cells

A human monocytic cell line (THP-1) was procured from ATCC and maintained in RPMI-1640 supplemented with 10% FBS, HEPES (10 mM), and *β*-mercaptoethanol (0.05 mM) at 37°C with 5% CO_2_. The THP-1 cells were dispersed on culture plates (2 × 10^5^ cells/mL) and treated with 25 nM phorbol 12-myristate 13-acetate (PMA) to obtain a M0 macrophage phenotype.

### 2.7. ELISA

The cell culture supernatant was collected, in which the concentrations of M1 cytokines IL-6, IL-1*β*, and TNF-*α* and M2 cytokines IL-10 and TGF-*β* were determined adhering to the instruction manual of the ELISA kits.

### 2.8. Transwell Assay

Cells were seeded to 24-well Matrigel-coated Transwell upper chambers at 5 × 10^4^ cells per well (200 *μ*L) and cultured in serum-free medium. Lower chambers were loaded with 800 *μ*L 10%FBS-contained medium. After 24 h, noninvading cells were discarded, and cells invaded through the lower membranes were fixed, stained with crystal violet, and counted under microscopy and analyzed by ImageJ.

### 2.9. Scratch Test

Cells were seeded in 24-well plates with serum-free medium. After cell adherence, the cell layer was scratched by a sterile pipette tip. The cell debris was rinsed away by PBS, and the width of scratch at 0 and 24 h was determined to evaluate the 24-h migration rate of cells by the following formula: migration rate = (width at 0 h − width at 24 h)/width at 0 h × 100%.

### 2.10. Flow Cytometry

After staining with phycoerythrin- or Alexa Flour488-conjugated antihuman or antimouse-specific antibodies, the expression of macrophage markers was analyzed by flow cytometry. All stained cells were examined by the flow cytometer (BD Bioscience, San Jose, CA, USA), and the data were analyzed by the Flow Jo (Tree Star, Ashland, OR, USA).

### 2.11. Immunofluorescence Staining

Cells were fixed with formaldehyde, penetrated with 0.5 Triton X-100, and blocked with 5% BSA for 1 h. After that, the cells were reacted with the primary antibodies overnight at 4°C and then with the goat antirabbit IgG (1 : 200, Thermo Fisher Scientific) at 37°C for 1 h. DAPI was used for nucleus staining. The staining was visualized and captured under a laser scanning confocal microscope.

### 2.12. Orthotopic Tumorigenesis in Mice

NOD/SCID mice (5 weeks old, 18.0-22.0 g) were procured from SLAC Laboratory Animal Co., Ltd. (Shanghai, China) and housed in standard conditions given rodent feed and drinking water ad libitum. Thereafter, 2 × 10^6^ stably transfected A549, or H125 cells were injected into the lung tissue of mice. After 6 weeks, the mice were sacrificed by overdosed pentobarbital (150 mg/kg) to harvest the tumor tissues. A half of each tumor tissue sample was digested for flow cytometry to examine the portion of F4/80/CD86- or CD206-positive cells. The other half of sample was used for immunohistochemistry (IHC) to determine the expression of CD86 and CD206.

The procedures for care and use of animals were approved by the Ethics Committee of the Fourth Affiliated Hospital of China Medical University, and all applicable institutional and governmental regulations concerning the ethical use of animals were followed.

### 2.13. Histological Staining

Tissue sections were dewaxed and loaded on glass slides for IHC. The tissue sections were treated xylene, rehydrated, treated with 3% H_2_O_2_, and blocked with 3% BSA for 1 h. Thereafter, the sections were covered with the antibodies at 4°C for 14 h and then with HRP-conjugated secondary antibody at 20-25°C for 3 h. After that, the sections were counter stained with hematoxylin, cleaned with xylene, and sealed for microscopy observation. The staining intensity of each tissue was scored. The IHC was scored from two aspects: the staining intensity (0, negative staining; 1, mild staining; 2, moderate staining; 3, strong staining) and the portion of positive cells (0, negative; 1, <10%; 2, ≥10% and<33%; 3,≥33% and<66%; and 4, ≥ 66%). The final IHC score was obtained by the product of the two scores above. A score of 6 was set as the cut-off value for genes with low (0-6) or high [6–12] expression.

### 2.14. Biotin-RNA Pull down

Biotinylated LINC01094 probe and the control probe were synthesized by the RioBio Co., Ltd. (Guangzhou, Guangdong, China). The RNA pull-down assay was then performed adhering to the manufacturer's instructions. The enrichment of target protein was analyzed by immunoblot analysis.

### 2.15. RNA Immunoprecipitation (RIP)

The RIP assay was carried out in accordance with the instruction manual of a Magna RIP kit (Millipore). The precipitated RNA was isolated by TRIzol and quantified by qPCR analysis after reverse transcription.

### 2.16. Chromatin Immunoprecipitation (ChIP)

Cells were fixed and soaked in formaldehyde (1%) for 10 min of DNA-protein cross-linking, and the reaction was terminated by glycine. The chromatin was ultrasonicated to 500-1,000 bp fragments. The ChIP assay was then performed following the instructions of a Magnetic Bead ChIP Kit (Thermo Fisher Scientific). The enriched DNA fragment was quantified by qPCR analysis.

### 2.17. Fluorescence In Situ Hybridization (FISH)

Cells were rinsed with RNase inhibitor-contained PBS for 3 min and treated with 0.3% Triton X-100 for 5 min. The cells were washed with washing buffer at 40°C, whereas tissue sections were rinsed with 2X SSC. The cell/tissue slides were reacted with preamplifier, amplifier, and labeling probe at 40°C for 1 h. After DAPI staining, the cells were observed under the microscope. For FISH assay, fixed cells were penetrated with 0.3% Triton-X 100, and sealed with 5% BSA. The tissue slides were soaked in acetone and treated soaked in the antigen retrieval solution at 37°C for 5 min. After hybridization with the FISH probe, the probes were washed, and the cells/tissue slides were incubated with the secondary antibody at 20-25°C for 1 h and then stained with 1 × DAPI. The images were captured under the confocal microscopy, and the colocalization in cells or tissues was analyzed by ImageJ.

### 2.18. Statistical Analysis

The SPSS 21.0 (IBM, SPSS, IL, USA) was used for data analysis. Data were presented as the mean ± SD. Differences between groups were analyzed by the Student's *t-*test or one-/two-way ANOVA. Kaplan-Meier analysis was conducted for data analysis. *p* < 0.05 refers to statistical significance.

## 3. Results

### 3.1. CCL7 Is Abundantly Expressed in LUAD and Linked to Increased TAM Infiltration

**Figure 1:**
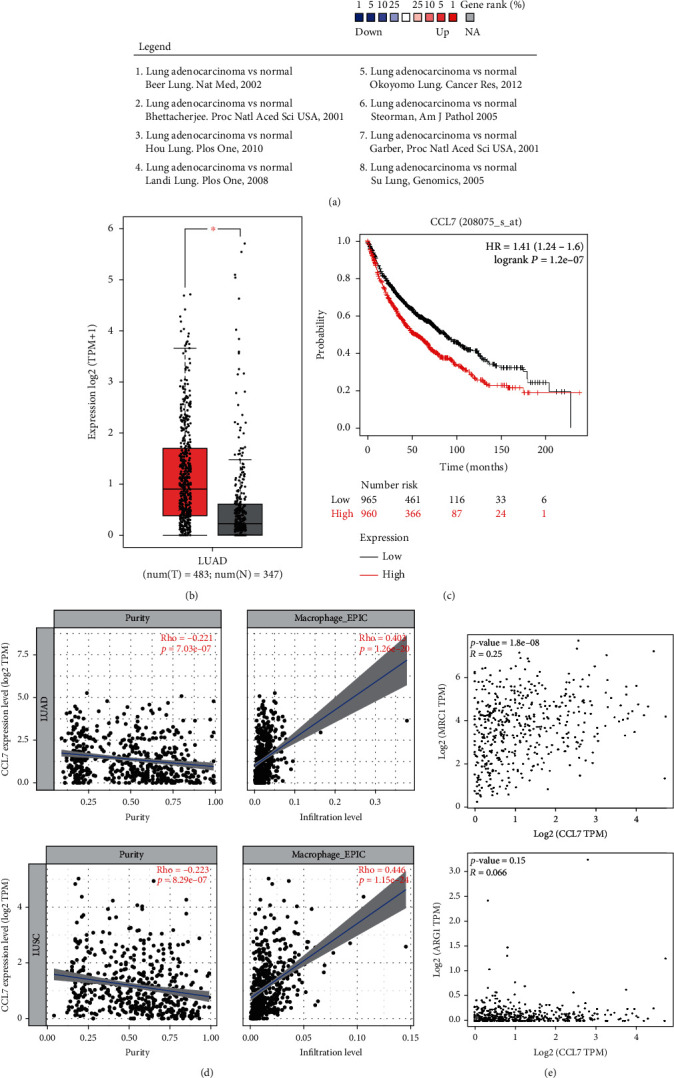
CCL7 is highly expressed in LUAD and linked to increased TAM infiltration. (a) CCL7 expression in LUAD and normal lung tissues in the Oncomine database; (b) CCL7 expression in TCGA-LUAD database of tumor tissues and in GTex database of normal lung tissues; (c) correlation between CCL7 expression and patient's prognosis; (d) correlation of CCL7 expression with the immune cell infiltration in LUAD patients; (e) correlations of CCL7 expression with the M2 macrophage markers ARG1 and CD206.

To examine the function of CCL7 in LUAD, we first predicted the CCL7 expression in several LUAD datasets in Oncomine (https://www.oncomine.org/). It was indicated that CCL7 expression is significantly increased in LUAD tissues versus the normal tissues (Figure 1(a)). A similar trend was observed in TCGA-LUAD (Figure 1(b)). Moreover, patients with higher CCL7 levels were suggested to have worse prognosis probability (Figure 1(c)). CCL7 is one of the chemokines that show close correlation with immunity. We therefore examined whether CCL7 has specific correlations with the immune cell infiltration in LUAD patients in TCGA-LUAD using the R CIBERSORT package. Of note, the CCL7 expression was found to be positively linked to macrophages infiltration (Figure 1(d)). In addition, data in TCGA-LUAD suggest that CCL7 have positive correlations with M2 macrophage markers Arg1 and CD206 (Figure 1(e)). Therefore, we postulated that CCL7 might affect the infiltration of M2 TAMs to promote LUAD development and lead to poor patient's prognosis.

### 3.2. CCL7 Knockdown Suppresses EMT and Mobility of LUAD Cells

**Figure 2:**
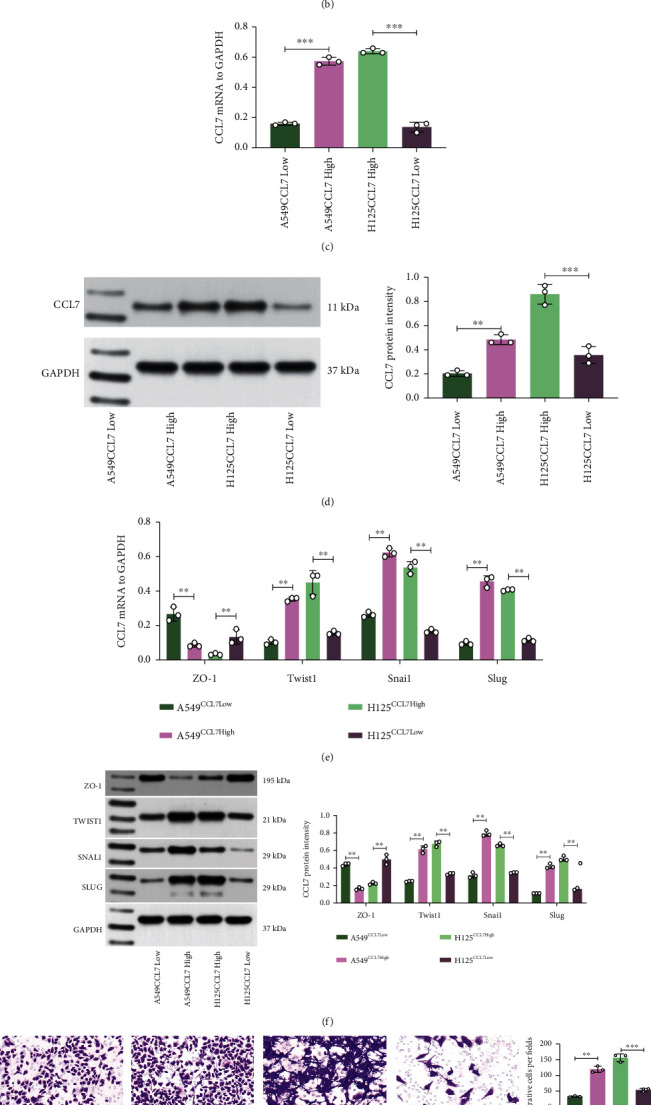
CCL7 knockdown suppresses EMT and mobility of LUAD cells. mRNA (a) and protein (b) levels of CCL7 in H1299, A549, H358 (low-invasive), and H125 (high-invasive) cells detected by qPCR and WB analyses; mRNA (c) and protein (d) levels of CCL7 in A549 and H125 cells after overexpression plasmid or shRNA transfection determined by qPCR and WB analyses; mRNA (e) and protein (f) levels of epithelial marker ZO-1 and mesenchymal markers Twist1, Snai1, and Slug in A549 and H125 cells examined by qPCR and WB analyses; (g) 24-h migration rate of A549 and H125 cells analyzed by the scratch test; (h) invasiveness of A549 and H125 cells analyzed by Transwell assay. Repetition = 3. ^∗∗^*p* < 0.01.

To confirm whether and how CCL7 affects LUAD progression, we first examined the CCL7 expression in H1299, A549, H358 (low-invasive), and H125 (high-invasive) cells. CCL7 had the lowest expression in A549 cells whereas the highest expression in H125 cells (Figures 2(a) and 2(b)). Thereafter, overexpression plasmid of CCL7 was transfected in A549 cells, while shRNA of CCL7 was transfected in H125 cells. The transfections were determined by qPCR and WB analyses, and the cells were designated A549^*CCL7*Low^ (transfected with empty plasmid), A549^*CCL7*High^ (transfected with CCL7 overexpression plasmid), H125^*Ccl7*high^ (transfected with scramble shRNA), and H125^*CCL7*Low^ (transfected with shRNA of CCL7), respectively (Figures 2(c) and 2(d)). Of note, we observed that the CCL7-low cells had significantly higher levels of ZO-1 (epithelial marker) whereas lower levels of Twist1, Snai1, and Slug (mesenchymal markers) than the CCL7-high cells (Figures 2(e) and 2(f)). In general, an increase in mesenchymal markers indicates a rise in the “mobility” of cells. Therefore, we further analyzed the mobility of cells using the scratch test and Transwell assay. It was observed that the CCL7-low cells had reduced migratory and invasive capacities compared to the CCL7-high cells (Figures 2(g) and 2(h)).

### 3.3. CCL7 Knockdown Suppresses Chemotactic Migration and M2 Polarization of Macrophages

**Figure 3:**
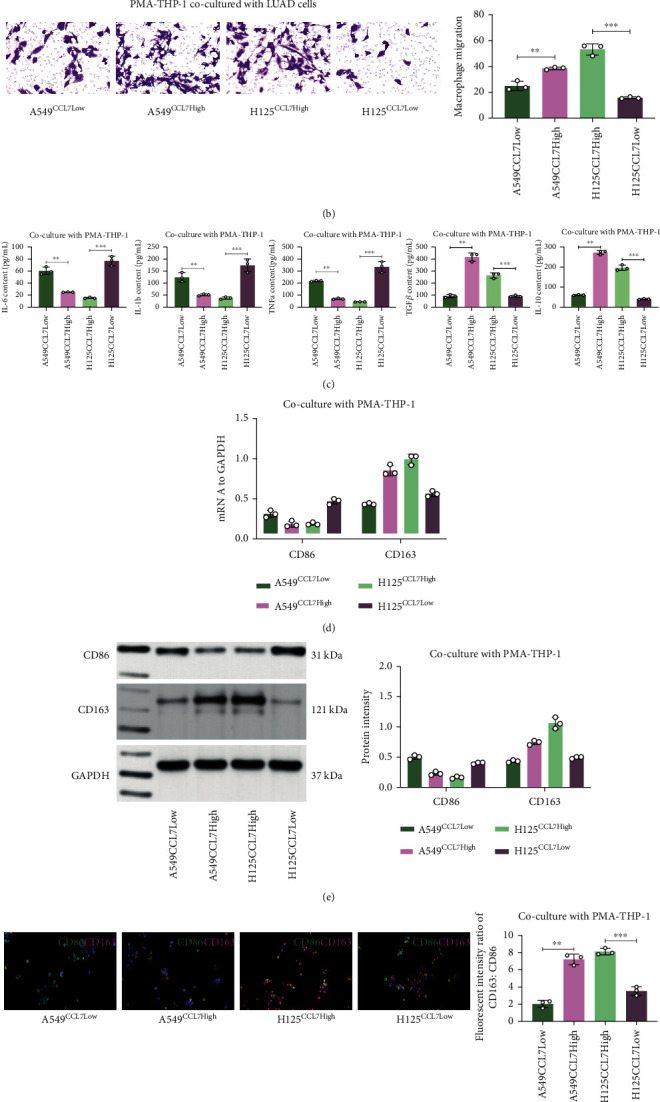
CCL7 knockdown suppresses chemotaxis and M2 skewing of macrophages. (a) A sketch map for the Transwell coculture system of PMA-treated THP-1 cells and LUAD cells (A549 and H125); (b) number of migrated THP-1 cells examined by crystal violet staining; (c) expression of M1 cytokines (IL-6, IL-1*β*, and TNF-*α*) and M2 cytokines (IL-10 and TGF-*β*) in the culture medium determined by ELISA kits; mRNA (d) and protein (e) levels of CD86 and CD206 in cocultured THP-1 cells examined by qPCR and WB analyses; (f) staining intensity of CD86 (red) and CD206 (green) in THP-1 cells determined by immunofluorescence staining. Repetition = 3. Data are presented as the mean ± SD. ^∗∗^*p* < 0.01.

The findings in Figure 2 indicates that high CCL7 expression is linked to the mobility of LUAD cells, and results in Figures 1(d) and 1(e) suggest that CCL7 is potentially linked to M2 macrophage infiltration. To test this, we treated THP-1 cells with PMA to induce M0 macrophages, which were then cocultured with A549 and H125 cells in Transwell chambers (Figure 3(a)). It was found that that the CCL7-high cells attracted increased number of macrophages compared to CCL7-low cells (Figure 3(b)). After that, we further examined the M1 cytokines IL-6, IL-1*β*, and TNF-*α* as well as M2 cytokines IL-10 and TGF-*β* in the culture supernatant. ELISA findings revealed that the THP-1 cells secreted increased M2 cytokines where reduced M1 cytokines when cocultured with the CCL7-high cells (Figure 3(c)). The THP-1 cells were harvested thereafter, and increased M2 markers along with decreased M1 markers were detected in macrophages cocultured with the CCL7-high LUAD cells, as examined by qPCR and WB analyses (Figures 3(d) and 3(e)). Moreover, the immunofluorescence assay further showed that the CD206 staining was strengthened whereas staining of CD86 was weakened in THP-1 cells cocultured with the CCL7-high LUAD cells (Figure 3(f)).

### 3.4. Xenograft Tumors Formed by CCL7-Low Cells Have Reduced Macrophage Infiltration

**Figure 4:**
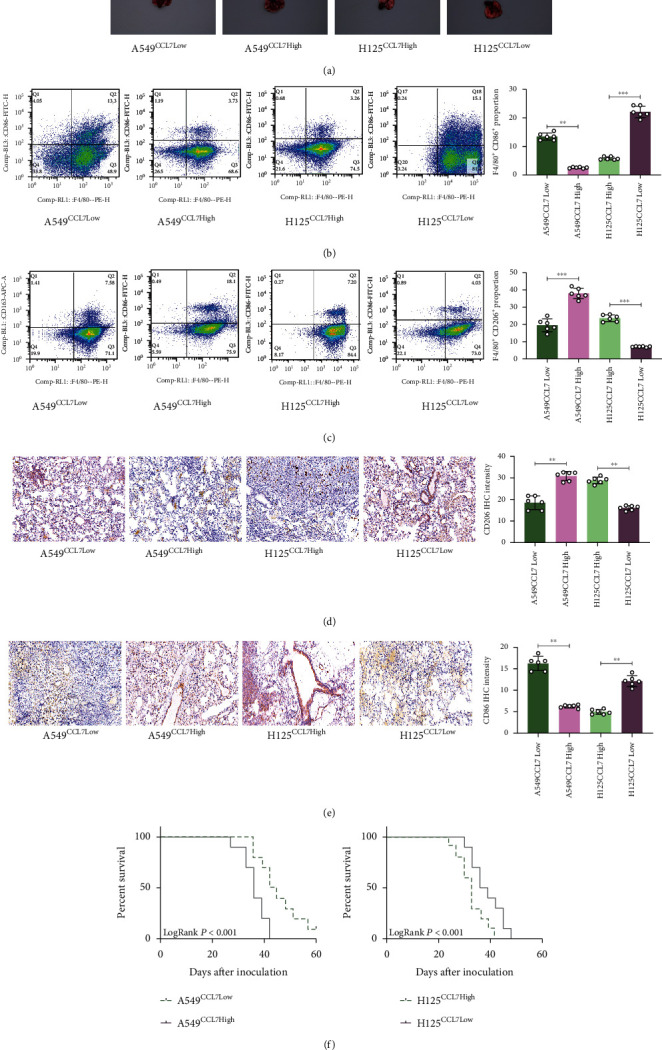
Xenograft tumors formed by CCL7-low cells have reduced macrophage infiltration. (a) Images of mouse lung tissues after animal sacrifice at 6 weeks after A549 or H125 cell injection; (b and c) portion of F4/80/CD86- or CD206-positive cells in mouse xenograft tumors analyzed by flow cytometry; (d and e) staining intensity of CD206 or CD86 in mouse xenograft tumors examined by IHC; (f) survival rate of another 10 mice in each group analyzed by Kaplan-Meier analysis. In panels (a–e), *n* = 6, in each group. ^∗∗^*p* < 0.01.

We then shifted the focus on the role of CCL7 in LUAD tumorigenesis *in vivo*. Stably transfected A549 or H125 cells were injected to the lung tissue of NOD/SCID mice. After 6 weeks, the animals were sacrificed to collect the lung tissues. It was found that the xenograft tumors formed by CCL7-low cells were in significantly smaller size than those formed by CCL7-high cells (Figure 4(a)). A half of each tumor tissue sample was digested for flow cytometry to examine the portion of F4/80/CD86- or CD206-positive cells. The other half of sample was used for IHC to determine the expression of CD86 and CD206. Both IHC and flow cytometry results revealed that the tumors formed by CCL7-low cells had significantly increased infiltration of M2 macrophages (Figures 4(b)–4(e)). Another 10 mice in each group were applied for survival test. It was observed that the survival rate of mice injected with CCL7-low cells was lower than those injected with CC7-high cells (Figure 4(f)). These findings indicate that CCL7 promotes LUAD tumorigenesis and M2 macrophage infiltration *in vivo* as well.

### 3.5. SPI1 Promotes CCL7 Transcription

**Figure 5:**
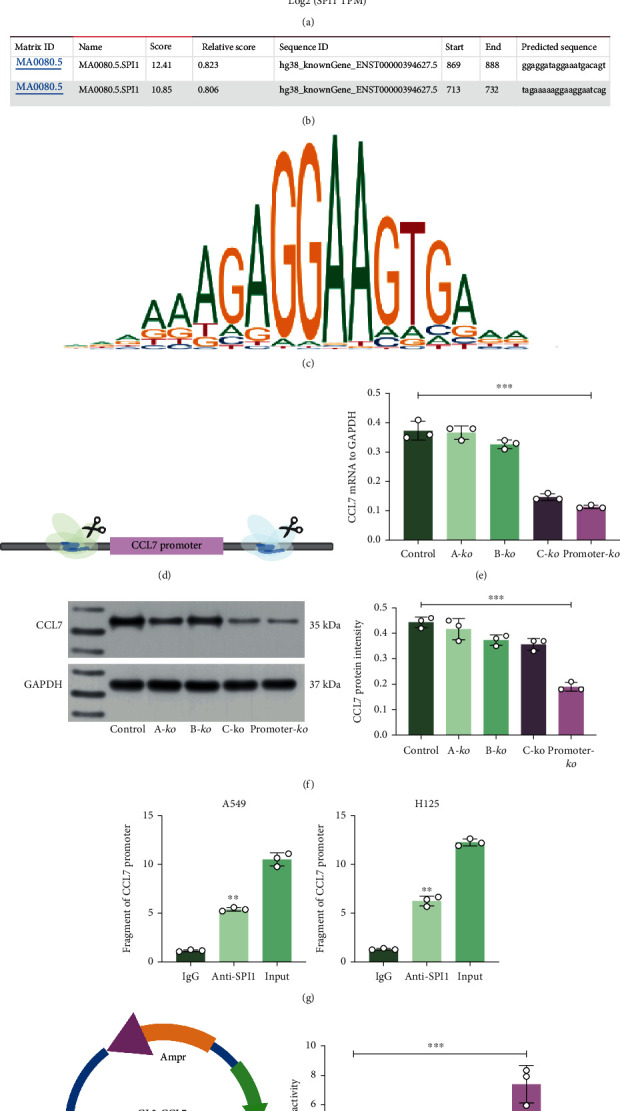
SPI1 promotes CCL7 transcription. (a) Transcription factors that can bind to CCL7 promoter with an over 0.5 correlation coefficient in TCGA-LUAD analyzed using the R TFBS package; (b) binding sites between SPI1 and CCL7 promoter; (c) conservative binding sequence of SPI1; (d) a sketch map for the fragmentation of CCL7 promoter sites using the Crispr-Cas9 system; mRNA (e) and protein (f) levels of CCL7 in A549 cells after knockout of A, B, and C fragments alone or simultaneously; (g) CCL7 promoter fragments enriched by anti-SPI1 examined by the ChIP-qPCR assay; (h) construction of the pGL4-Luc luciferase vector containing the CCL7 promoter sequence for the examination of SPI1-CCL7 binding by luciferase assay. Repetition = 3. Data are presented as the mean ± SD. ^∗∗^*p* < 0.01.

To find the possible mechanisms responsible for CCL7 upregulation in LUAD, we then analyzed the transcription factors that can bind to CCL7 promoter with an over 0.5 correlation coefficient in TCGA-LUAD using the R TFBS package. SPI1 was the only outcome showing a correlation coefficient of 0.58 with the CCL7 promoter (Figure 5(a)). Thereafter, we further analyzed the binding between SPI1 and CCL7 promoter via JASPAR (http://jaspar.genereg.net/). We first obtained from Ensembl (http://www.ensembl.org/index.html) that the promoter sequence of CCL7 is located at chr17:34,269,621-34,270,220. According to the prediction results, we found that SPI1 have two main binding sites with CCL7 promoter (Figures 5(b) and 5(c)). To clarify the specific binding sequence, the promoter sites were fragmented to three sections A, B, and C) using the Crispr-Cas9 system (Figure 5(d)). It was noteworthy that the CCL7 expression in A549 cells was reduced after knockdown of the fragment C or fragments A, B, and C; however, the gene expression was not significantly affected in the absence of A or B fragments (Figures 5(e) and 5(f)). ChIP-qPCR assay was then performed to examine the binding between SPI1 and CCL7. It was found that the abundance of CCL7 promoters reacted with anti-SPI1 was significantly increased compared to IgG (Figure 5(g)). In the luciferase assay, the CCL7 promoter sequence was inserted to the pGL4-Luc vectors, which was cotransfected with overexpression plasmid of SPI1 in 293 T cells. The luciferase activity in cells was increased upon SPI1 overexpression (Figure 5(h)). Therefore, it can be opined that SPI1 binds to CCL7 promoter to activate its activation.

### 3.6. Overexpression of SPI1 in CCL7-Low Cells Promotes Macrophage Migration and M2 Polarization

**Figure 6:**
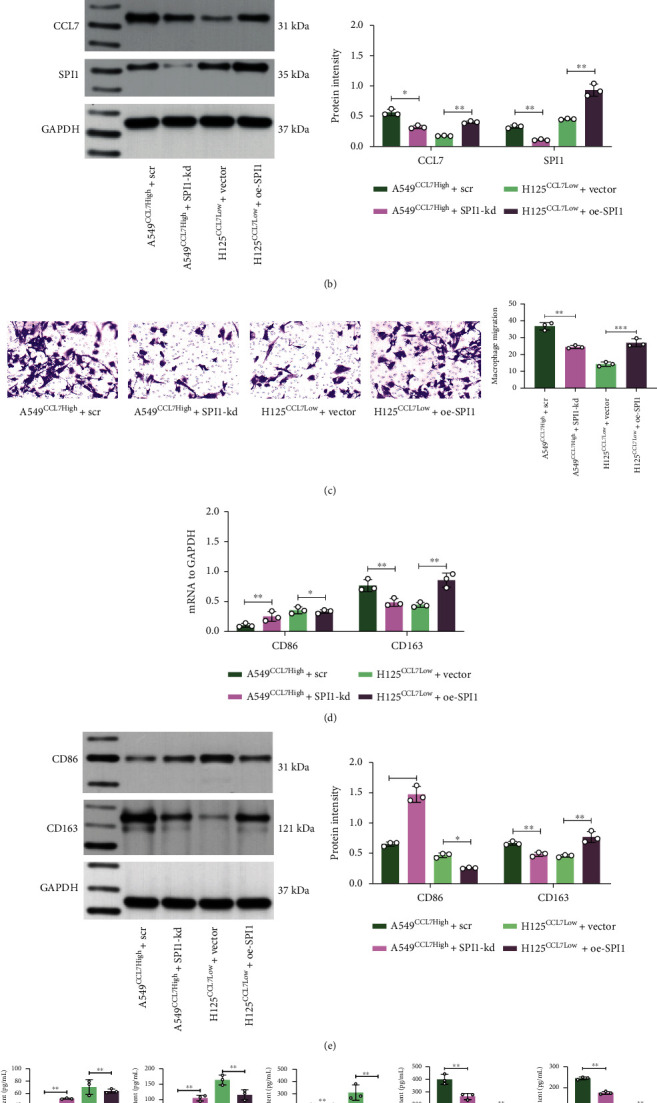
Overexpression of SPI1 in CCL7-low cells promotes macrophage migration and M2 polarization. mRNA (a) and protein (b) levels of SPI1 and CCL7 in H125^*CCL7*Low^ and A549^*CCL7*High^ cells after SPI1 overexpression plasmid or shRNA transfection detected by qPCR and WB analyses; (c) number of migrated THP-1 cells examined by crystal violet staining; mRNA (d) and protein (e) levels of CD86 and CD206 in cocultured THP-1 cells examined by qPCR and WB analyses; (f) expression of M1 cytokines (IL-6, IL-1*β*, and TNF-*α*) and M2 cytokines (IL-10 and TGF-*β*) in the culture medium determined by ELISA kits. Repetition = 3. Data are presented as the mean ± SD. ^∗∗^*p* < 0.01.

To validate the functional interaction between SPI1 and CCL7, we transfected overexpression plasmid of SPI1 in H125^*Ccl7*Low^ cells and shRNA of SPI1 in A549^*CCL7*High^ cells. The overexpression of SPI1 restored the CCL7 expression in H125 cells and vice versa (Figures 6(a) and 6(b)). These cells were cocultured with PMA-treated THP-1 cells in the Transwell system as well. Of note, the chemotactic migration of THP-1 cells was promoted when cocultured with H125^*Ccl7*Low^ cells upon SPI1 upregulation but suppressed with A549^*CCL7*High^ cells upon SPI1 downregulation (Figure 6(c)). The polarization of THP-1 cells was analyzed as well. As expected, the SPI1-overexpression condition promoted the M2 macrophage polarization as well as the secretion of M2 cytokines. Reverse trends were found in the setting of SPI1 suppression where the M2 skewing of THP-1 cells was significantly blocked (Figures 6(d)–6(f)).

### 3.7. LINC01094 Binds to SPI1 to Regulate CCL7 Expression

**Figure 7:**
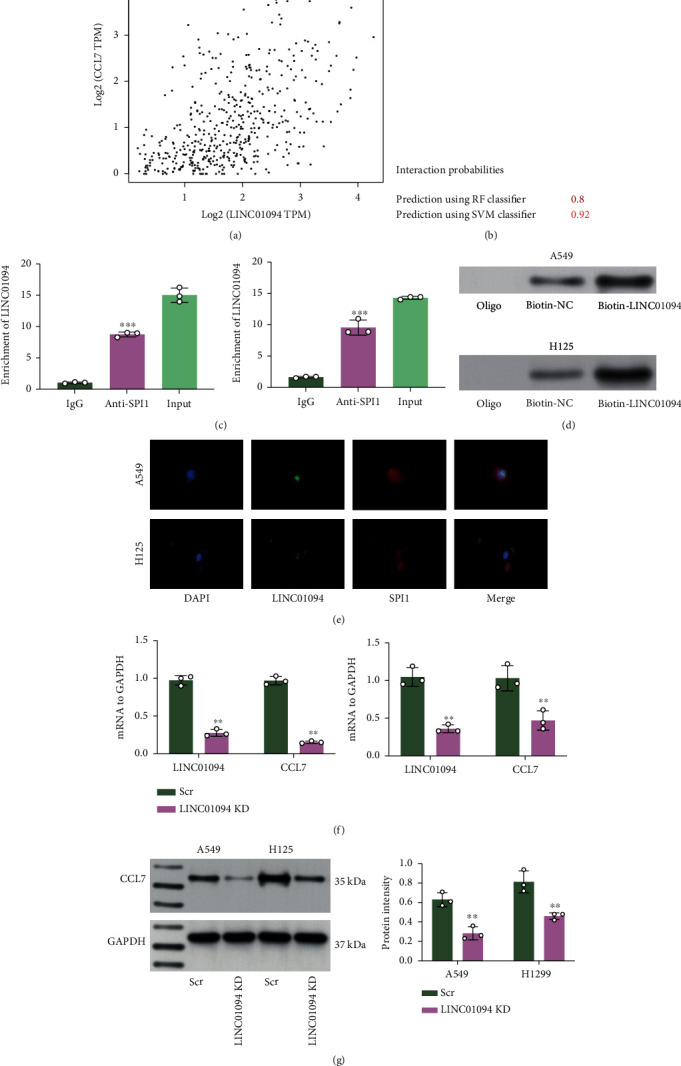
LINC01094 binds to SPI1 to regulate CCL7 expression. (a) Correlation of LINC01094 with CCL7 predicted in the TCGA-LUAD database; (b) binding relationship between LINC01094 and SPI1 validated in TCGA-LUAD database; binding between LINC01094 and SPI1 examined by RIP (c) and biotin-LINC01094-based RNA pull down (d) assays; (e) subcellular localization of LINC01094 and SPI1 in H125 and A549 cells examined by FISH assay; mRNA (f) and protein (g) levels of CCL7 in A549 and H125 cells after LINC01094 knockdown examined by qPCR and WB analyses. Repetition = 3. Data are presented as the mean ± SD. ^∗∗^*p* < 0.01.

More molecules involved in CCL7 regulation were explored. We obtained 510 tumor samples from TCGA-LUAD and discarded over 80% nonexpressed genes with Log2 (TPM+1) as the thresholds. Moreover, according to the cor. test and Pearson's correlation analyses, we obtained that LINC01094 showed strong correlation with CCL7 (Figure 7(a)). Moreover, data in the RPIseq system suggested that LINC01094 has significant binding relationship with SPI1 (Figure 7(b)). To validate this, we performed RIP assay, by which we found increased LINC01094 enrichment in the complexes precipitated by anti-SPI1 (Figure 7(c)). Moreover, the biotin-LINC01094-based RNA pull-down assay also suggested that LINC01094 could bind to SPI1 (Figure 7(d)). The FISH assay also suggested that LINC01094 and SPI1 had a colocalization in nucleus (Figure 7(e)). To confirm the potential relationship between LINC01094 and CCL7, artificial knockdown of LINC01094 was introduced in A549 and H125 cells, after which the CCL7 expression was significantly reduced as well (Figures 7(f) and 7(g)). Taken together, we opine that LINC01094 binds to SPI1 and promotes its nuclear translocation, which therefore binds to CCL7 promoter and activates its transcription, leading to increased chemotaxis and M2 skewing of macrophages in LUAD and aggravated tumor progression.

## 4. Discussion

In the TME, the major population of TAM is M2 phenotype which expresses immune checkpoint modulators including programmed death ligand 1 to grant immunosuppression to cancer cells; however, they may reskew to the M1 phenotype and fulfill tumor-suppressing functions [9, 15]. Identifying key molecules implicated in the M2 polarization of macrophages may help develop novel therapeutic targets for tumor elimination. In the present study, we observed that CCL7, which can be upregulated by LINC01094-mediated SPI1, is linked to increased tumor M2 macrophage accumulation and malignant development of LUAD cells.

CCL7 has reportedly been highly expressed in advanced cancers and linked to the more aggressive malignant phenotype of cancer cells such as survival, proliferation, EMT, invasion, and metastasis [16]. Likewise, CCL7 upregulation has been observed in the NSCLC tissues in the study by Han et al., and this overexpression was linked to poor prognosis of patients [14]. In this work, we first predicted elevated CCL7 expression in LUAD and its correlation with increased macrophage infiltration using bioinformatics tools involving Oncomine and TCGA-LUAD. Later, we identified increased CCL7 expression and observed that the CCL7 knockdown in LUAD cells suppressed the EMT and metastasis of CC. More importantly, the CCL7 knockdown blocked the chemotaxis and M2 polarization of macrophages. In a previous report by Parikh *et al.*, CCL7 was identified in lung adenomas extracted from aged mice with significant immune cell accumulation, and the adenomas from these mice showed higher invasiveness [17]. Moreover, high CCL7 levels recruit the TAMs expressing CCR2 on their surface, leading to increased vascular permeability [18, 19]. Similar situations have been found in NSCLC that CCL7 recruited TAM to the tumors via interacting its receptors including CCR2, CCR3, CCR4, and CCR5 to augment its malignant development [20]. Interestingly, CCL7 has also been identified as a chemoattractant for neutrophils participating in the TME formation, which increased the invasiveness of cells [21]. In this work, the promoting roles of CCL7 in M2 skewing and tumor development were validated *in vivo* that the CCL7 suppression blocked the xenograft tumorigenic ability of cells and suppressed M2 macrophage infiltration in the tumor tissues.

When it comes to the upstream regulator of CCL7 in LUAD, we obtained the transcription factor SPI1 as its regulator via bioinformatics analyses and luciferase and immunoprecipitation assays. SPI1 is located in the p11.22 region of human chromosome 11 [22]. SPI1 fulfills critical functions in the maintenance of immune cells, with its knockout or defects leading to defects in multiple types of immune cells including macrophages [23, 24]. A recent publication by Huang *et al.* revealed that SPI1 was correlated with a multitude of infiltrating immune cells and tumor-related signaling pathways, therefore, leading to poor prognosis for gastric cancer [25]. Likewise, SPI1 has been identified as one of the prognostic genes for esophageal squamous cell carcinoma correlating with M2 macrophage accumulation and poor patient's prognosis [26, 27]. SPI1 was reported as a candidate gene for squamous lung cancer [28]. It has been reported to activate the transcription of SnoRNA host gene 6 to promote the growth and mobility of NSCLC cells [29]. However, there has no evidence concerning the role of SPI1 in the macrophage skewing and accumulation in lung cancer. In this work, we found that the M2 skewing of macrophages blocked by CCL7 knockdown was restored upon SPI1 overexpression. Moreover, we identified that lncRNA LINC01094 showed a positive correlation with SPI1 via having binding relationship with SPI1. Though without protein-coding functions, the lncRNAs fulfill significant functions in biology via RNA-DNA, RNA-RNA, or RNA-protein interactions [30]. They can bind with specific transcription factors to achieve gene regulation at the transcriptional level [31]. The LINC01094 has been linked to development and progression of several cancer types [32, 33], though its specific role in lung cancer remains undefined yet. More importantly, this lncRNA has been found to trigger EMT of gastric cancer cells via modulating macrophage infiltration. Here, we found that LINC01094 could bind to SPI1 to enhance its nuclear translocation and transcriptional function, which is possibly responsible for the CCL7 upregulation in LUAD.

Collectively, this research suggests that LINC01094 binds to SPI1 to promote its nuclear translocation, which further activates CCL7 transcription by binding to its promoter, leading to M2 macrophage accumulation and dissemination of tumor cells. However, one major weak point of the present work is that the exact roles of LINC01094 were not investigated. More experiments concerning the relevance of LINC01094 to macrophage infiltration and LUAD development, both *in vitro* and *in vivo*, are required in the future. Targeting any member of the LINC01094/SPI1/CCL7 axis may help reduce the infiltration of M2 macrophage and metastasis of LUAD cells.

## Figures and Tables

**Table 1:** Catalogue numbers of the ELISA kits.

Target	Cat. no
IL-1*β*	QK201
IL-6	QK206
TNF-*α*	QK210
IL-10	D1000B
TGF-*β*	DB100B

Note: ELISA: enzyme-linked immunosorbent assay; IL: interleukin; TNF: tumor necrosis factor; all kits were procured from the R&D Systems Inc. (Minneapolis, MN, USA).

**Table 2:** Primer sequences for qPCR analysis.

Symbol	Forward (5′-3′)	Reverse (5′-3′)
CCL7	ACAGAAGGACCACCAGTAGCCA	GGTGCTTCATAAAGTCCTGGACC
ZO-1	GTCCAGAATCTCGGAAAAGTGCC	CTTTCAGCGCACCATACCAACC
TWIST1	GCCAGGTACATCGACTTCCTCT	TCCATCCTCCAGACCGAGAAGG
SNAI1	TGCCCTCAAGATGCACATCCGA	GGGACAGGAGAAGGGCTTCTC
SLUG	ATCTGCGGCAAGGCGTTTTCCA	GAGCCCTCAGATTTGACCTGTC
CD86	CCATCAGCTTGTCTGTTTCATTCC	GCTGTAATCCAAGGAATGTGGTC
CD163	CCAGAAGGAACTTGTAGCCACAG	CAGGCACCAAGCGTTTTGAGCT
SPI1	GACACGGATCTATACCAACGCC	CCGTGAAGTTGTTCTCGGCGAA
LINC01094	GCCAGCCTAAGGAACACGTA	GAGTTCAAAGGGCCCCCATC

**Table 3:** Antibodies, dilution rates, the catalogue numbers, and the manufacturers.

Antibodies	Dilution	Cat. no	Manufacture
CCL7	1 : 1,000	#MA5-29089	Thermo Fisher Scientific
ZO-1	1 : 2,000	#40-2300	Thermo Fisher Scientific
TWIST1	1 : 500	ab50887	Abcam
SNAI1	1 : 2,000	GTX125918	Genetex
SLUG	1 : 500	ab27568	Abcam
CD86	1 : 1,000	ab239075	Abcam
CD206	1 : 500	GTX42264	Genetex
SPI1	1 : 2,000	#PA5-17505	Thermo Fisher Scientific
GAPDH	1:5,000	ab8245	Abcam
F4/80	1 : 1,000	GTX26640	Genetex

## Data Availability

The authors confirm that the data supporting the findings of this study are available from the corresponding author on reasonable request.

## Supplementary Materials

Supplementary Materials Supplementary file 1: full scans of original gels presented in this study.
