# Supplementary figures and images for: LINC01094/SPI1/CCL7 Axis Promotes Macrophage Accumulation in Lung Adenocarcinoma and Tumor Cell Dissemination
Source: J Immunol Res. 2022 Sep 9;2022:6450721. doi: 10.1155/2022/6450721 (PMC9481385; doi:10.1155/2022/6450721)

2B

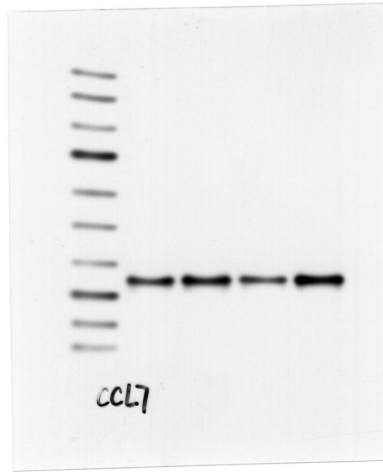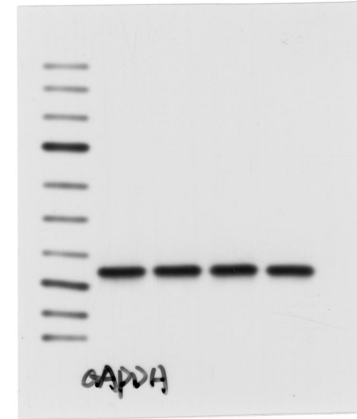

2D

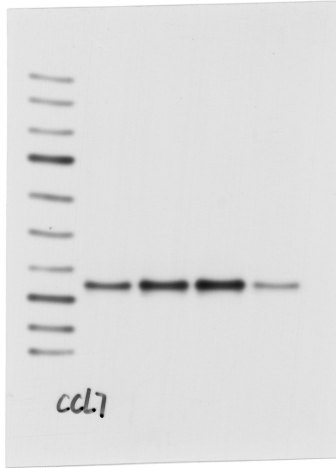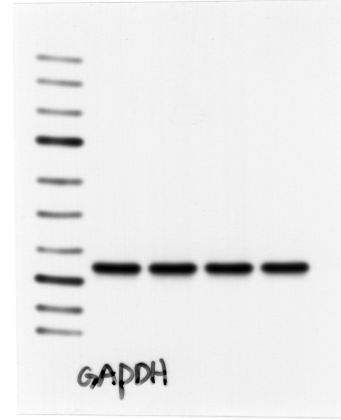

2F

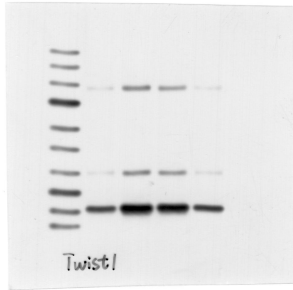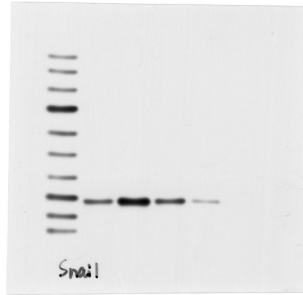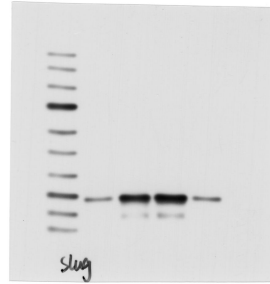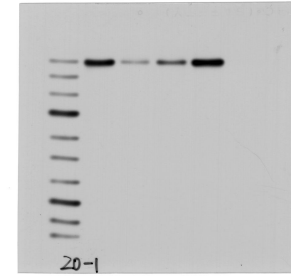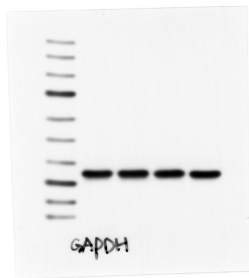

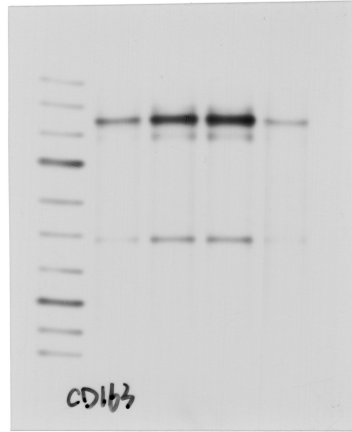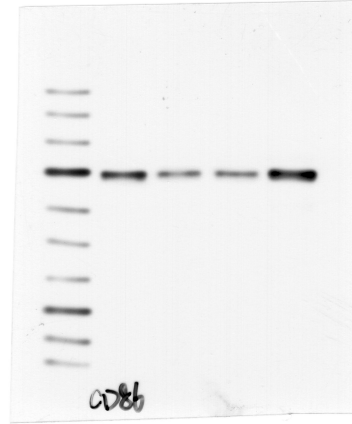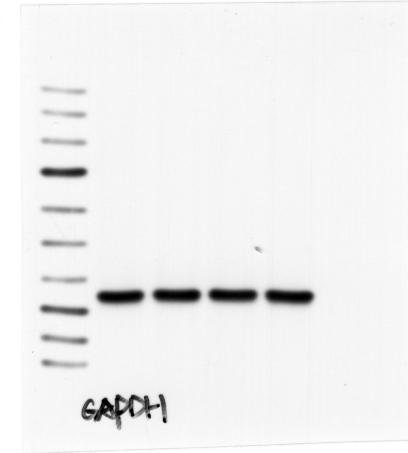

5F

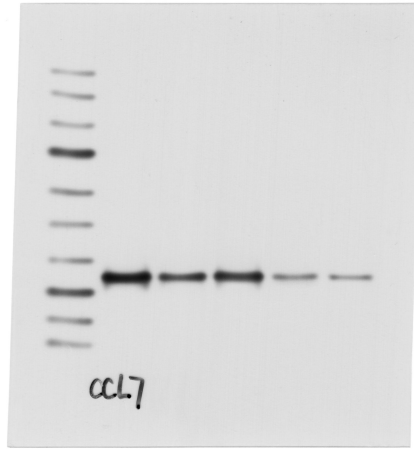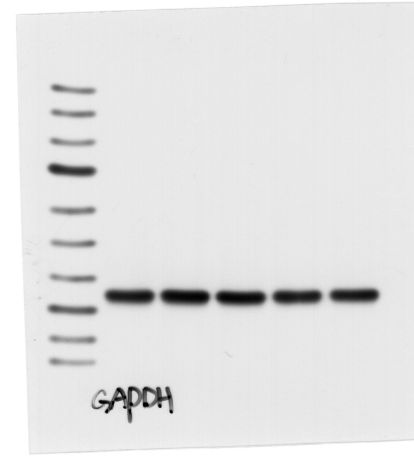

6B

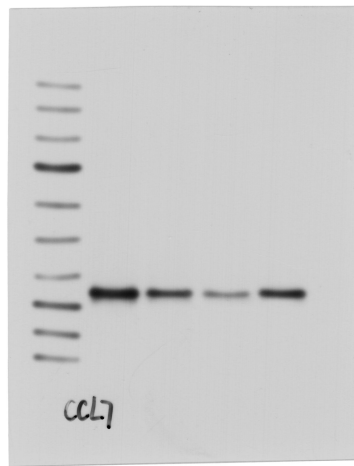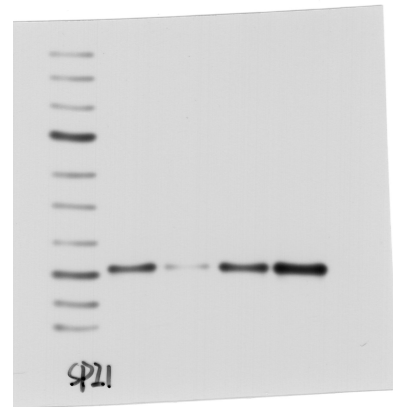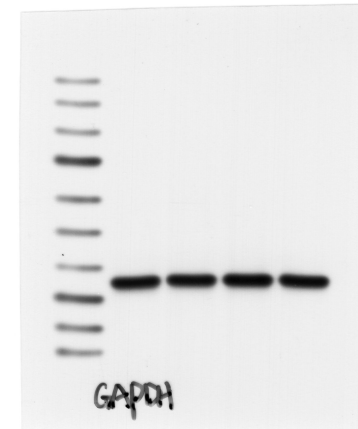

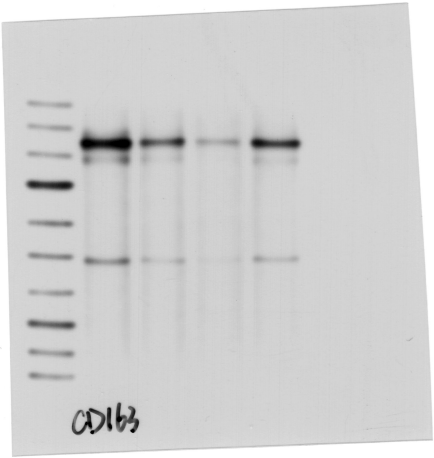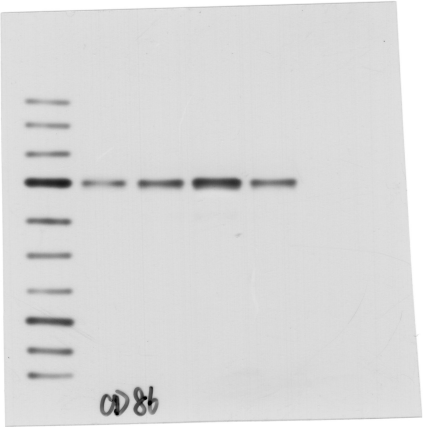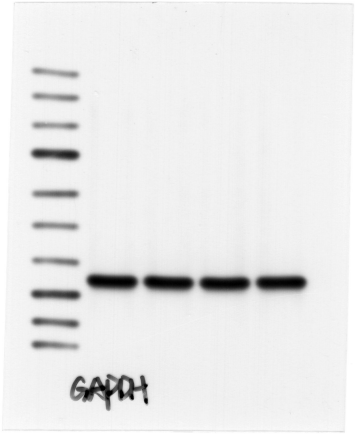

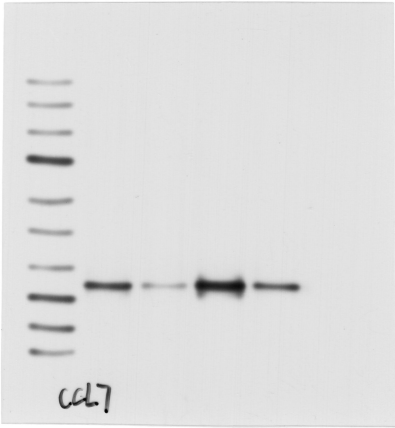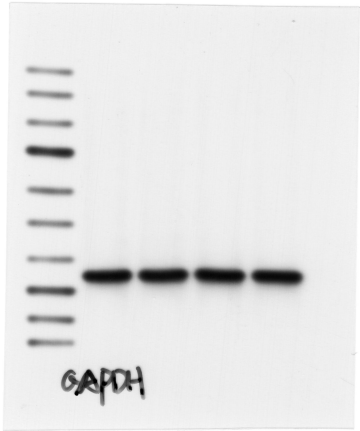

Supplement: Supplementary Materials — Supplementary file 1: full scans of original gels presented in this study. [file 6450721.f1.pdf]
